# Effects of Eribulin on Epithelial–Mesenchymal Plasticity in Patient-Derived Breast Cancer Cultures and Excised Tissues

**DOI:** 10.3390/cancers18040598

**Published:** 2026-02-11

**Authors:** Charles L. Bidgood, Erika Morera, Binny Jaradi, Tirsa van Wyngaard, Anu T. Koikalethu, Nathalie Bock, Veenoo Agarwal, Andrew D. Redfern, Erik W. Thompson

**Affiliations:** 1 Centre for Genomics and Personalised Health, School of Biomedical Sciences, Faculty of Health, Queensland University of Technology, Brisbane, QLD 4059, Australia; 2 Translational Research Institute, Brisbane, QLD 4102, Australia; 3 Centre for Biomedical Technologies, School of Biomedical Sciences, Faculty of Health, Queensland University of Technology, Brisbane, QLD 4059, Australia; 4 Department of Breast and Endocrine Surgery, Princess Alexandra Hospital, Woolloongabba, QLD 4102, Australia; 5 Max Planck Queensland Centre, Brisbane, QLD 4000, Australia; 6 Medical School, University of Western Australia, Crawley, WA 6009, Australiaandrew.redfern@health.wa.gov.au (A.D.R.)

**Keywords:** breast cancer, eribulin, EMT, chemoresistance

## Abstract

Background: The cytotoxic agent eribulin has been shown to promote EMT reversion, reduce treatment resistance, and potentially enhance responses to a range of therapeutic agents. Methods: We examined the effects of eribulin in patient-derived breast cancer tissues and a phenotypically heterogeneous breast cancer cell line to assess EMT and chemotherapy response. Results: Nanostring-based analysis of EMT-associated gene expression in breast cancers from patients receiving standard-of-care TAC neoadjuvant chemotherapy compared to a cohort receiving neoadjuvant eribulin (NeoEribulin trial) showed markedly reduced expression of EMT markers in cancers treated with eribulin versus TAC. Through single-cell immunofluorescent imaging and analysis, we identified that HCC38 triple-negative breast cancer (TNBC) cells exhibited a shift towards an epithelial-like state marked by E-cadherin upregulation following acute eribulin exposure, but not with other chemotherapeutic agents. Investigation of primary breast cancer cultures derived from pre-neoadjuvant biopsies also revealed that HER2-enriched primary breast cancer cells displayed heightened chemosensitisation to doxorubicin (Adriamycin), which was not recapitulated in TNBC cultures. Conclusions: Our data highlight the presence of contextual parameters which govern the degree of EMT regulation by eribulin.

## Simple Summary

Eribulin is an approved therapy for the treatment of breast cancer which has been shown to reverse the epithelial-to-mesenchymal transition (EMT) and improve the efficacy of standard chemotherapies in cell lines, animal studies, and clinical specimens. Tumour EMT status has also been linked to eribulin efficacy. Based on this, we evaluated the effects of eribulin in patient-derived breast cancer cultures and a triple-negative breast cancer cell line to assess changes to EMT and therapy response. We further identified the induction of epithelial-like characteristics, including E-cadherin expression in a patient-derived HER2+ primary tissue with a predominantly mesenchymal phenotype following longitudinal eribulin exposure. Additionally, we compared EMT marker expression in breast cancers treated with standard-of-care neoadjuvant docetaxel, Adriamycin and cyclophosphamide (TAC) therapy with that observed in the neoadjuvant eribulin clinical trial.

## 1. Introduction

The epithelial-to-mesenchymal transition (EMT) is a cellular program in which polarized epithelial cells lose defining characteristics such as a compact morphology and cell-to-cell adhesion to adopt mesenchymal-like properties such as enhanced motility and migratory capacity [1]. Transient activation of a partial, hybrid, or complete EMT is a major driver of cellular plasticity in cancer, contributing to key factors affecting mortality such as oncogenesis, the development of metastases, and treatment resistance—particularly to chemotherapies [2]. The ability to therapeutically modulate epithelial–mesenchymal plasticity (EMP) provides an exciting avenue to improve patient survival and clinical outcomes in a wide range of malignancies.

EMT may also hinder the success of immunotherapy by altering critical immune checkpoint molecules, tumour antigen processing and presentation [3], and expression of immunomodulatory factors [4,5]. In breast cancer, there is established evidence for the efficacy of immunotherapy in TNBC [6], but many tumours in other sub-types, particularly the most common luminal A sub-type, are immunologically cold with respect to current immunotherapies so that patients do not benefit. Therefore, engineered phenotypic shifts along the epithelial–mesenchymal axis could improve the success of cancer immunotherapy strategies. Eribulin has been shown to favourably influence immunosensitivity, including by increasing cytotoxic T-cells and reducing regulatory T-cells in breast cancer patients, changes that correlate with higher response rates [7], making it a suitable candidate for further immunomodulatory research.

Extensive data link tumour hypoxia to aggressive behaviour and enhanced dissemination [8]. Bevacizumab, a treatment that inhibits new blood vessel formation, was found to increase initial response rates to chemotherapy but failed to improve survival, likely due to the resulting tumour hypoxia produced and consequent accelerated progression [9]. Hypoxia is also a well-established driver of EMT, with vascular targeting by bevacizumab also inducing mesenchymal shift [10]. In contrast, eribulin has been shown to increase tumour vessel density and maturation with consequent improved perfusion. This can reverse EMT as well as promote delivery of other therapeutic agents [11]. Eribulin induced maturation of vascular pericytes and promoted vessel normalization in mouse models and eribulin-treated patients, accompanied by improved tumour immune infiltration and enhanced immunotherapy responses and survival in the mouse model [12].

Tumour stroma has been demonstrated to frequently contain fibroblasts with alterative phenotypic features to those in benign stroma, termed cancer-associated fibroblasts (CAFs). Extensive work has demonstrated that CAFs can substantially contribute to cancer progression [13]. Mechanistically, studies across multiple tumour types implicate CAFs in the induction of EMT within the tumour [14]. Again, eribulin has shown the ability to normalize the phenotype of CAFs, causing reversion to a benign stromal expression profile, simulating the impact of inhibition of the classical EMT-driving TGF-β pathway [15].

Eribulin is a potent cytotoxic agent approved for breast cancer and liposarcoma treatment [16,17]. It has been shown to reverse EMT, a process associated with tumour progression, metastasis, and therapy resistance [2,18], introducing the possibility that eribulin could have broader chemosensitising benefits to other agents beyond its direct cytotoxic action. Eribulin can reverse EMT in a broad range of pre-clinical contexts, including TGFβ- and chemotherapy-induced EMT in breast cancer cell lines in vitro and in vivo [19,20,21], as well as EMT caused by infection of immortalized oral keratinocytes with latent Kaposi’s sarcoma-associated herpesvirus and human papillomavirus 31 [22]. In clinical studies, eribulin has also been shown to cause elevated epithelial:mesenchymal protein ratios in recurrent or metastatic breast cancers [23]. This contrasts with most other cytotoxic drug classes that induce EMT [24]. The reversal of EMT by eribulin in clinical trials has been associated with increased overall survival in comparison with other therapies [25], beyond that attributable to direct cytotoxicity and the initial disease control period.

The objective of our study is to assess the ability of eribulin to differentially modulate EMP and chemosensitivity in models which closely capture the underlying heterogeneity and clinical presentation of EMT in breast cancer. To accomplish this, we first analysed transcriptomic profiles of tumours from breast cancer patients who had received neoadjuvant eribulin versus neoadjuvant standard chemotherapy to assess differences in canonical epithelial versus mesenchymal gene expression. To resolve acute EMT responses on a cell-by-cell basis, we applied a single cell immunofluorescent imaging workflow to an inherently EMT-heterogeneous breast cancer cell line. We then employed patient-derived breast cancer cultures spanning clinical receptor types to test whether eribulin alters sensitivity to doxorubicin, a key chemotherapy within the standard TAC regime in clinically relevant tissues. Finally, longitudinal immunofluorescent profiling of EMT markers was performed in a HER2+ primary breast cancer culture to characterize temporal changes in EMT-associated proteins and cellular morphology.

Our findings support the ability of eribulin to exert a cytotoxic effect without the induction of EMT, compared to standard anthracycline and taxane cytotoxics, and show the potential for eribulin to supress constitutive EMT and promote chemosensitisation in some, but not all, breast cancers. Understanding the contextual basis for this will have far-reaching implications for individualizing treatment regimens to enhance breast cancer therapy responses. It is important to understand that this response is not constitutive, and that identification of E/M state biomarkers will be required to guide usage and timing of companion treatments to allow translation.

## 2. Materials and Methods

### 2.1. Cell Line Culture

HCC38 cells (triple negative breast cancer cell line), known to exhibit EMP [26] were obtained from the ATCC and maintained in RPMI 1640 (Gibco, ThermoFisher Scientific, Waltham, MA, USA) supplemented with 10% foetal bovine serum (Gibco, ThermoFisher Scientific, MA, USA) at 37 °C/5% CO_2_. All cells were passaged, and their medium replenished every 48–72 h. Cells were routinely screened for mycoplasma using the Translational Research Institute’s mycoplasma testing service. To observe and quantify acute changes to EMT following high-dose anti-neoplastic drug treatment, HCC38 cells were exposed to eribulin (1 nM, ERI), doxorubicin (1 μM, DOX), docetaxel (10 μM, DOC), paclitaxel (10 μM), 4-hydroperoxy-cyclophosphamide (50 μM), carboplatin (CPN, 10 μM), cisplatin (10 μM), olaparib (100 μM) or vehicle control (DMSO) for 24 h prior to immunofluorescent staining (Section 2.5) and single-cell image analysis (Section 2.6).

### 2.2. Patient-Derived Cultures and Treatment

Breast cancer patient-derived cultures were established with human ethics approval (ethics number HREC/2020/QRBW/61294) and informed consent, as previously described [27], with the exception that they were derived from biopsies taken prior to neoadjuvant chemotherapy (NACT). Tumour characteristics, clinical subtypes, and prior treatment status are provided in Table 1. For CBCa51, we also confirmed that estrogen receptor (ER) expression was retained following long-term culture (Supplementary Figure S1). To determine changes to chemosensitivity following eribulin treatment, patient-derived cultures were subject to dose–response analysis (Section 2.4). To observe acute and sustained changes to EMT in patient culture CBCa50, longitudinal eribulin treatment was also performed for 0, 1, 3, 5 and 7 days prior to immunofluorescent staining (Section 2.5).

### 2.3. Comparison of Gene Expression Changes Seen After Neoadjuvant Therapy

We compared gene expression changes from the NeoEribulin trial [28] in which locally advanced breast cancer patients received neoadjuvant eribulin (data kindly provided by SOLTI Breast Cancer Research Group, Barcelona, Spain) and our own cohort of 30 breast cancer patients treated with standard-of-care [29]. These patients were selected from the Medical Oncology Department database at a single institution, Royal Perth Hospital (RPH), Australia. They were all treated between 2001 and 2010 with neoadjuvant anthracycline and taxane and had been similarly consented at initial post-diagnostic assessment for tissue usage. The study was approved by the RPH Human Research Ethics Committee, approval number RGS0000001981 (HREC: 2013-130). Core biopsies and post-treatment surgical specimens were available on all 30 patients. The characteristics of this patient cohort are shown in Table 2. Pathologic complete response (pCR) was achieved in seven patients (23%).

We developed a 250-gene Nanostring assay comprising 130 markers from a range of EMT studies across diverse biological systems and 120 breast cancer contextual markers. This combined probe set was validated using RNA extracted from MDA-MB-468 cells stimulated for EMT by EGF or hypoxia [30]. Pre- and post-TAC NACT formalin-fixed and paraffin-embedded (FFPE) samples were biopsied for RNA extraction and Nanostring analysis as previously described [31,32]. Fifty of these genes profiled were represented in the NeoEribulin trial (SOLTI-1007, NCT01669252) and could be compared to neoadjuvant TAC within the current study. Statistical significance was determined using the two-stage linear step-up procedure of Benjamini, Krieger and Yekutieli, with Q = 5% between fold change values calculated from each cohort. The complete statistical summary is available in Supplementary Table S1.

### 2.4. Dose–Response Analysis

Dose–response analysis was performed on primary patient cultures using the CellTitreGlo ATP-based viability reagent (Promega, Madison, WI, USA). Cells were pre-treated for seven days with eribulin (0.5 nM) or vehicle control (DMSO). Eribulin and vehicle was also replenished after 72 h to account for drug degradation in culture. Cells were then exposed to a range of concentrations of doxorubicin, outlined in each relevant figure, for 72 h prior to endpoint using the CellTitreGlo assay. For TNBC cultures, this was also conducted with paclitaxel, docetaxel, and 4-hydroperoxy-cyclophosphamide, an active metabolite of cyclophosphamide, to compare differences in chemosensitivity profiles. Cell viability was then calculated from luminescent readings obtained from the ClarioStar Plus (BMG-labtech, Ortenberg, Germany). Dose–response analysis and calculation of IC_50_ values were determined with GraphPad Prism 10 using the [inhibitor] vs. normalized response (variable slope) non-linear fit model.

### 2.5. Immunofluorescence Microscopy

Patient-derived CBCa50 cells and the HCC38 cell line were seeded in 96-well optical imaging plates (CellVis P96-1.5H-N) and subject to the experimental conditions outlined in both Section 2.1 and Section 2.2. At endpoint, medium was removed and the plate was washed twice with 200 µL PBS (and in-between each step). Cells were fixed with 4% paraformaldehyde (Electron Microscopy Sciences, Hatfield, PA, USA) at room temperature for 15 minutes and then permeabilized with 0.4% Triton X for 10 min (Sigma Aldrich, Burlington, MA, USA). Cells were blocked in 5% (*w*/*v*) bovine serum albumin (BSA) for 1 h at room temperature prior to incubation with directly conjugated primary antibodies against: Vimentin (Santa Cruz Biotechnology, Heidelberg, Germany; SC-6260); Pan-Cytokeratin (Invitrogen, ThermoFisher Scientific, Waltham, MA, USA; 41-9003-82); or E-cadherin (BioLegend, San Diego, CA, USA; 324112) in blocking buffer. Plates were imaged on the Operetta CLS system (Revvity, Waltham, MA, USA) at the Translational Research Institute (Brisbane, Australia) Microscopy Facility.

### 2.6. HCC38 Single-Cell Imaging and Analysis

Single-cell imaging analysis was used to quantify EMT on a cell-by-cell basis and to identify cell populations perturbed by each investigated antineoplastic therapy. Images acquired following immunofluorescent staining of HCC38 cells were subject to a custom image analysis workflow which involves cellular segmentation by Cellpose [33] and subsequent feature extraction of fluorescent signal and morphometric parameters with scikit-image [34]. The resultant feature matrix was processed by principal component analysis and subjected to non-linear dimension reduction by Uniform Manifold Approximation and Projection (UMAP). Unsupervised clustering was accomplished using the Leiden algorithm [35] and data visualization accomplished with the matplotlib and seaborn python libraries [36,37].

## 3. Results

### 3.1. Eribulin Is Associated with Muted EMT Induction Compared to Standard-of-Care Chemotherapy

**Figure 1:**
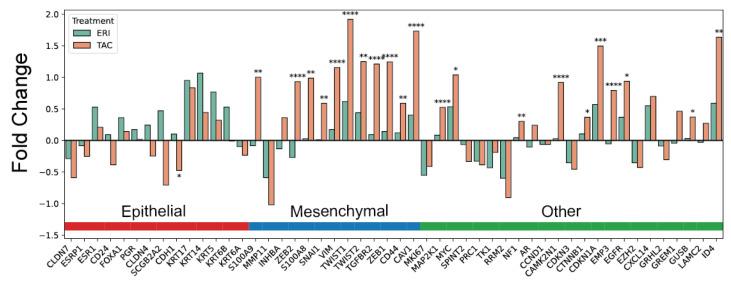
Eribulin is associated with a muted EMT compared to other anti-neoplastic agents. Nanostring-derived gene expression changes following neoadjuvant eribulin (ERI) compared to standard-of-care neoadjuvant docetaxel, Adriamycin and cyclophosphamide (TAC) in patient breast tumours. Statistical significance was determined using the two-stage linear step-up procedure of Benjamini, Krieger and Yekutieli, with Q = 5% between fold change values calculated from each cohort, ns–not significant, * *p* < 0.05, ** *p* < 0.01, *** *p* < 0.001, **** *p*  <  0.0001.

To investigate the clinical relevance of (EMT) in patients treated with eribulin, we compared two Nanostring expression–based datasets derived from patient tumours that had undergone neoadjuvant docetaxel, Adriamycin, and cyclophosphamide (TAC) or neoadjuvant eribulin (NeoEribulin trial [28]). Tumours from patients treated with TAC (Table 2) showed a statistically significant upregulation of canonical EMT markers and transcription factors associated with a mesenchymal cell state, including *ZEB1*, *ZEB2*, *TWIST1*, *TWIST2*, *VIM*, and *SNAI1*, when compared with those treated with eribulin (Figure 1, Supplementary Table S1). In contrast, *CDH1* (E-cadherin) was significantly downregulated following TAC treatment, indicating a loss of epithelial characteristics, whereas eribulin treatment was not associated with *CDH1* downregulation. Notably, other EMT-associated genes, *CAV1*, *CD44*, and *EGFR*, were also among the significantly upregulated genes following TAC treatment. Interestingly, both eribulin and TAC treatment were associated with increased expression of epithelial cytokeratins (*KRT17*, *KRT14*, *KRT5*, and *KRT6B*), as reflected by increased fold change, with no statistically significant difference between the two treatment groups. Collectively, these data indicate that, unlike standard chemotherapy, eribulin is not associated with substantial EMT activation and supports the possibility of an eribulin-mediated mesenchymal-to-epithelial transition (MET).

**Figure 2:**
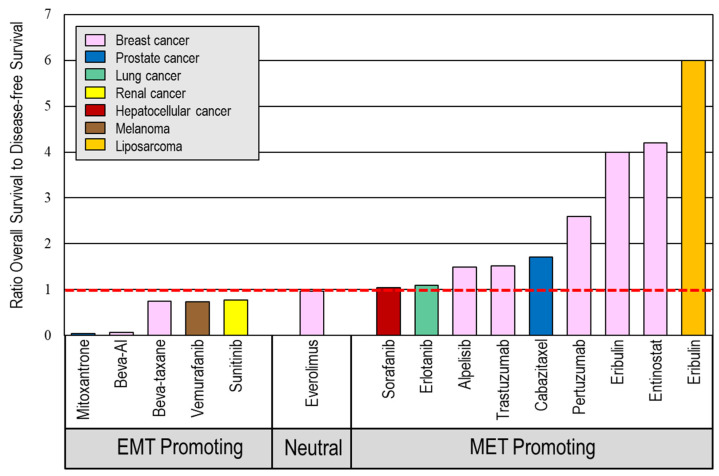
A comparison of overall survival and progression-free survival (disease control) benefits for a range of therapeutic agents in randomized clinical trials according to their impact on EMT. (Beva–bevacizumab, AI–aromatase inhibitor). A ratio below 1.0 indicates that the overall survival benefit is shorter than the increase in disease control period implying accelerated cancer progression after treatment completion. A ratio above 1.0 indicates extended survival beyond that of disease control implying an inhibition of cancer progression after treatment completion.

Our team also previously explored whether certain treatments shown to stimulate either EMT or MET [24] correlated with a loss of initial treatment benefit in later disease based on both pre-clinical (EMT) and clinical (survival data) evidence. Following on from this, we adapted this analysis to include several other cancers and clinical studies not previously shown (Supplementary Table S2). Collectively, therapies with evidence of EMT-inducing capabilities tend to benefit progression-free survival, while therapies with EMT-reverting (or MET-activating) capabilities tend to benefit overall survival (Figure 2). Notably, eribulin was associated with one of the largest progression-free-survival-to-overall-survival benefit ratios compared to other MET-driving therapies in both liposarcoma and, importantly, breast cancer. This supports the idea that EMT-inducing drugs tend to accelerate disease progression in cancers following their initial survival benefit, while EMT-repressing drugs tend to have an inverse relationship.

### 3.2. Single-Cell Immunofluorescent Imaging Reveals Altered EMT Cell Population Dynamics Following Acute Eribulin Exposure in TNBC

**Figure 3:**
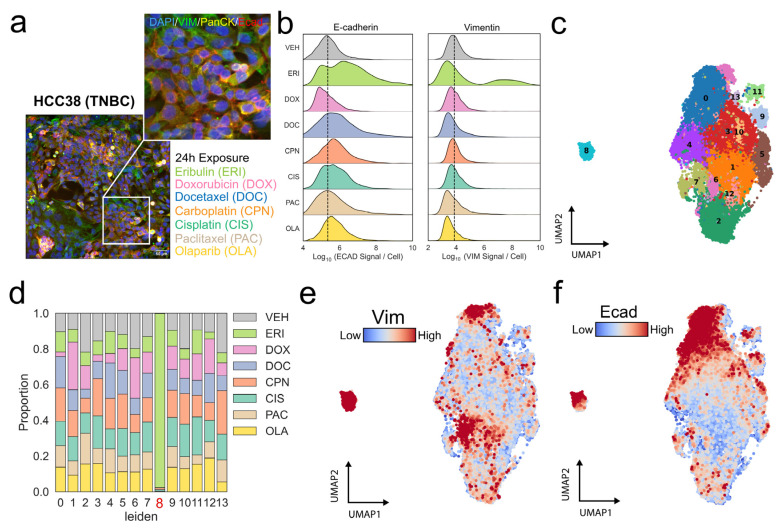
Immunofluorescence profiling and single-cell imaging reveals EMT perturbation following acute exposure to eribulin compared to standard chemotherapies in TNBC. (**a**) Immunofluorescence staining of vimentin (green), Pan-cytokeratin (yellow) and E-cadherin (red) in the HCC38 TNBC cell line. A magnified region of interest (white box) has been included to show underlying E/M heterogeneity and list of color-coded drugs denoted beneath, scale = 50 µm. (**b**) Smoothed density plot by kernel density estimation (KDE) of log-transformed immunofluorescent signals per cell of E-cadherin (**left**) and vimentin (**right**) across vehicle control (DMSO), standard chemotherapies and olaparib. The dashed-line indicates mean of vehicle control (VEH) (**c**) Dimension reduction and by UMAP and subsequent Leiden-based unsupervised clustering of HCC38 cells (*n* = 27,241). Cluster identity is overlayed on top of each computed cluster (0–13). (**d**) Proportion of cells in each cluster annotated by drug which they were exposed to, Cluster 8 (red) predominantly consists of cells treated with Eribulin. Log-transformed immunofluorescent signal of (**e**) Vimentin (Vim) and (**f**) E-Cadherin (Ecad) overlayed on previously computed UMAP embeddings.

To investigate the short-term effects of eribulin (24 h) compared with other chemotherapies on EMT, HCC38 TNBC cells were exposed to a panel of antineoplastic agents, including eribulin. HCC38 cells were selected due to their intrinsic heterogeneity and the presence of dual cell populations occupying either epithelial or mesenchymal states, as defined by mixed expression of vimentin, pan-cytokeratin, and E-cadherin (Figure 3a). Quantitative, multiparametric single-cell segmentation was performed to extract EMT-related protein expression and morphometric features on a per-cell basis. Whereas most antineoplastic agents induced only minor perturbations in E-cadherin or vimentin expression, eribulin exposure resulted in increased E-cadherin expression within a distinct subpopulation of cells. Notably, a small subset of cells also exhibited increased vimentin signal following eribulin treatment (Figure 3b). Dimensionality reduction using UMAP of the multiparametric imaging features followed by unsupervised clustering with the Leiden algorithm identified discrete cell populations across the dataset (Figure 3c). A distinct cluster (Cluster 8) was identified, that consisted almost exclusively of eribulin-exposed cells (Figure 3d). However, eribulin-treated cells were not solely confined to Cluster 8 and were also distributed across other clusters, indicating that only a subpopulation of eribulin-exposed cells exhibited this response. Further characterization of Cluster 8 revealed concomitantly high expression of both E-cadherin and vimentin relative to the primary cell cluster (Figure 3e,f).

**Figure 4:**
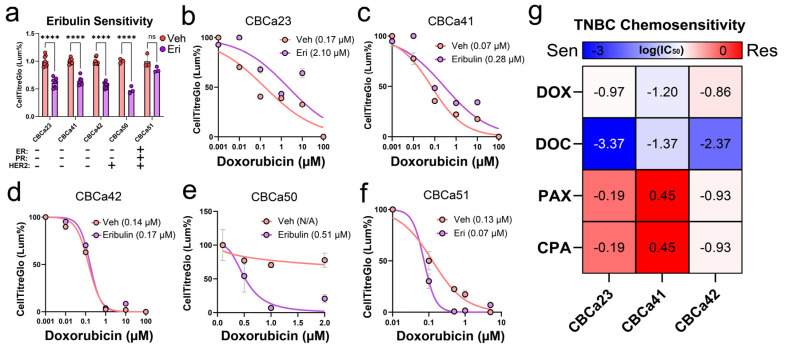
Eribulin and chemosensitivity profiles of primary breast cancer cultures. (**a**) Comparison of patient-derived primary culture sensitivity to eribulin at 0.5 nM determined by ATP-based viability (CellTitreGlo), technical replicates range between 3 and 8 for each sample due to variable amount of material obtainable from primary samples. (**b**–**f**) ATP-based viability (CellTitreGlo) dose–response profiles of CBCa23, 41, 42 (ER−/PR−/HER2−), CBCa50 (ER−/PR−/HER2+) and CBCa51 (ER+/PR+/HER2+) to doxorubicin following 7 days of pre-exposure to vehicle control (Veh) or 0.5 nM eribulin mesylate (Eri). Half-maximal inhibitory concentrations log_10_(IC50) are shown adjacent to the figure legend text in brackets. (**g**) Heatmap detailing differences in log_10_(IC50) derived chemosensitivity profiles of TNBC primary cells against doxorubicin (DOX), docetaxel (DOC), paclitaxel (PAX) and 4-hydroperoxy cyclophosphamide (CPA). Statistical significance determined by two-way ANOVA with Sidak’s multiple comparisons test, ns—not significant, **** *p*  <  0.0001.

Eribulin has been previously demonstrated to chemosensitise breast tumours, most likely attributed to its established ability to revert EMT [19,38]. We established multiple primary breast cancer cultures derived from patient biopsies taken prior to neoadjuvant chemotherapy to investigate the effect of eribulin on doxorubicin (Adriamycin) sensitivity, a key therapy employed in standard high-risk adjuvant therapy regimen. Firstly, we compared three primary TNBC, and two HER2-enriched cultures (one triple-positive and one HER2+ only) for stand-alone eribulin sensitivity using a standardized dose (0.5 nM). We found that while TNBC cultures (CBCa23, 41, 42) and the HER2+ culture (CBCa50) shared similar sensitivity to eribulin exposure, the triple-positive case (CBCa51) was less sensitive, with no significant differences to the vehicle control (Figure 4a). It should also be noted that while CBCa51 had the greatest mean response to eribulin, it was only significantly different from one of the TNBC cases (CBCa41, Supplementary Figure S2). In the same experiment, we exposed each culture to seven days of eribulin (0.5 nM) or vehicle, and then compared their resultant half-maximal inhibitory concentrations (IC_50_) to doxorubicin. This analysis revealed that the non-TNBC cultures displayed heighted sensitivity to doxorubicin, particularly the ER−/PR−/HER2+ CBCa50 line, while the TNBC cases displayed no change or a minimal reduction in chemoresistance (Figure 4b–f). While no significant changes to chemosensitivity were observed in the TNBC cases, we also compared chemosensitivity profiles of these cases against doxorubicin (DOX), docetaxel (DOC), paclitaxel (PAC) and 4-hydroperoxy-cyclophosmide (CPA, active mimetic). While eribulin sensitivity was previously identified to be largely conserved amongst all three cultures, unique chemosensitivity profiles were uncovered with respect to other agents for each case (Figure 4g).

**Figure 5:**
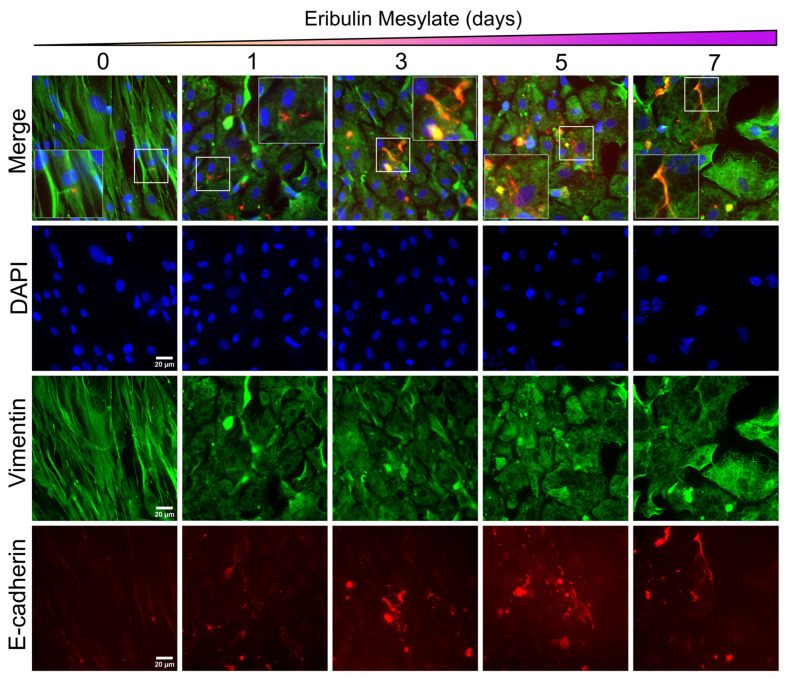
Eribulin induces E-cadherin expression and disrupts intermediate filament organization in primary HER2+ breast cancer cells. Immunofluorescent staining of vimentin (green) and E-cadherin (red) in primary patient-derived breast cancer cells (Case #CBCa50, ER−/PR−/HER2+) following 1, 3, 5 and 7 days of exposure to eribulin (0.5 nM), compared to vehicle control (DMSO, day 0). Induction of E-cadherin expression is observable as early as day 1 and remains sustained throughout the treatment period. Magnified region of interest indicated by white box, scale = 20 µm.

Given our observation of chemosensitisation of HER2+ primary breast cancer cells (CBCa50) to doxorubicin, we next investigated whether longitudinal exposure to eribulin was associated with alterations in EMP. Immunofluorescence analysis of untreated CBCa50 cells demonstrated a pronounced mesenchymal phenotype at baseline, characterized by an elongated cellular morphology and minimal E-cadherin expression (Figure 5). Following 24 h of eribulin exposure, marked morphological changes were observed, including a transition from an elongated, mesenchymal-like morphology to a more cuboidal, epithelial-like appearance. Consistent with these morphological alterations, E-cadherin expression became detectable after 24 h of treatment and was maintained at 3, 5, and 7 days of continuous exposure. In contrast, vimentin expression was not reduced over the course of the experiment. However, a clear change in vimentin subcellular organization was evident, with a shift from a structured, aligned filamentous pattern in untreated cells to a more disrupted and dispersed distribution following eribulin treatment.

**Figure 6:**
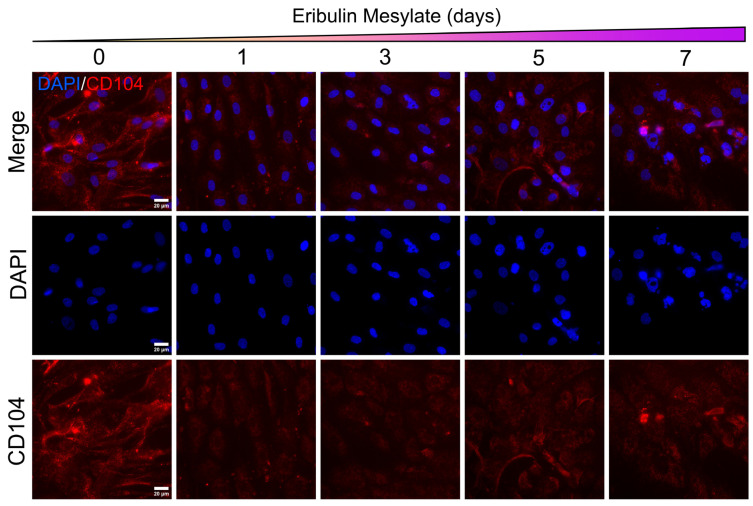
Eribulin reduces CD104 (ITGB4) expression in primary HER2+ breast cancer cells. Immunofluorescent staining of CD104 (ITGB4, red) in primary patient-derived breast cancer cells (Case #CBCa50, ER−/PR−/HER2+) following 1, 3, 5 and 7 days of exposure with eribulin (0.5 nM), compared to vehicle control (DMSO, day 0). Reduction in CD104 expression is observable as early as day 1 and remains sustained throughout the treatment duration. Scale = 20 µm.

CD104 (integrin β4; ITGB4) has been identified as a marker of hybrid epithelial–mesenchymal (hybrid E/M) cell populations and has been associated with cancer cell stem-like behaviour [39,40]. Baseline immunofluorescence analysis revealed moderate CD104 expression in CBCa50 cells, consistent with a partial EMT or hybrid-E/M phenotype, in agreement with our prior observations of high vimentin and low E-cadherin expression. Using the same experimental conditions described above, CBCa50 cells exhibited a marked reduction in CD104 expression following 24 h of eribulin exposure. This loss of CD104 was maintained with continued treatment at 3, 5, and 7 days (Figure 6). Notably, the reduction in CD104 coincided temporally with the emergence of epithelial-like morphological features and induction of E-cadherin expression, suggesting a coordinated shift in epithelial–mesenchymal marker expression following eribulin treatment.

## 4. Discussion

There are several studies showing the effects of eribulin, primarily in its ability to induce epithelial phenotypes [2,19,38]. In our study, we highlight a reduction in EMT-related features across clinical specimens and cell lines. We saw evidence of this in the HCC38 TNBC cell line, which exhibited significant *de novo* epithelial–mesenchymal plasticity [26], as evident in Figure 3a, and responded strongly both morphologically and in epithelial gene expression to eribulin. Similarly, the HER2+ CBCa50 patient-derived culture showed a prominent increase in E-cadherin expression and adopted cobblestone cell morphology (Figure 5). Although the levels of expression of vimentin remained high, the protein lost filamentous form, which would be expected to affect the functionality or regulation of the protein in migration and invasion, important mechanisms during EMT [41,42,43].

Furthermore, Bagheri and colleagues observed a consistent reduction in the EMT score in breast tumours after neoadjuvant treatment with eribulin compared to TAC. This was based on the multiplexed immunohistochemistry for Snail1, Zeb1, Keratin-8, Keratin-14, vimentin and E-cadherin, compared to the parallel study of TAC NACT where the EMT score remained high [38]. The attenuation of EMT features was largely due to a decrease in cells expressing vimentin and an increase in cells expressing Keratin-8 (an established luminal phenotype marker) or Keratin-8 plus E-cadherin, rather than proportional changes in E/M-hybrid phenotype of cellular expressing E-cadherin and vimentin. The SOLITI group that undertook the NeoEribulin trial also reported clinical shifts from basal to normal-like subtypes, which would be consistent with reduced EMT, importantly, in addition to the shift from luminal-B to luminal-A also reported in their studies [28].

These phenotypic changes may underpin the improved clinical benefit of eribulin, evidenced by the prolongation of overall survival to a greater extent than the progression-free survival period seen on treatment (Figure 2) [44,45]. A reduction in new metastases has been observed, rather than effects on established metastases [25], consistent with an EMT-reversal action suppressing metastatic spread. As shown above (Figure 2), other cancer therapies that categorically reverse EMT (i.e., cause MET, e.g., eribulin, cetuximab and entinostat) also show extended survival benefits in clinical trials beyond on-treatment disease control periods. This contrasts with the majority of current chemotherapies, hormonal therapies, and targeted agents that cause EMT [24], where overall survival benefits are often disappointingly short despite initially encouraging on-treatment control periods, suggestive of accelerated progression following treatment cessation.

It is interesting to consider the observations seen clinically—that mesenchymal positioning on the EMT axis has been associated with better eribulin response. Mesenchymal differentiation of the primary tumour, indicated by reduced E-cadherin or increased vimentin immunostaining, was associated with eribulin response in metastatic breast cancer cases [46]. Chan et al., in 2024, further showed that ZEB1 overexpression in metastatic samples had shorter times to recurrence, while high expression levels for SNAIL1 or TWIST1 in the primary BC were associated with significantly longer survival in patients who received eribulin [47]. They also showed that high vimentin levels were associated with a clinically relevant trend toward shorter survival after CDK4/6 inhibition. Although we did not assess this quantitatively, we did see rather consistent potent responses of our different patient-derived cultures to eribulin at 0.5 nM (established as typical IC_50_), with the exception of CBCa51 (Figure 4f). Importantly, sublethal concentrations of eribulin in CBCa51 were sufficient to chemosensitise them to doxorubicin.

Although we saw appreciable chemosensitisation after eribulin pre-treatment in CBCa50 and CBCa51 cells, this was not universally seen in the other patient-derived cultures. It was somewhat surprising that the chemosensitisation by eribulin was evident in the ER and/or HER2 positive lines compared to the TNBC samples CBCa23, CBCa41 and CBCa42. Enhancement of therapy response to other agents has been seen with in vitro synergism between eribulin and 5-fluoruracil (5-FU) in TNBC models in vitro and in vivo [20,48], and in mouse (PB3) and human (MDA-MB-231) mammary cancer cells [38]. Eribulin also sensitized oral squamous carcinoma cells to cetuximab therapy via induction of MET [21]. Similarly, the combination of the PI3K inhibitor alpelisib with eribulin more effectively suppressed the growth of paclitaxel-resistant cells in vitro and in vivo than alpelisib alone [49]. In a pre-clinical model of ovarian carcinosarcoma, where the sarcomatous component is characterized by EMT [50], eribulin produced more potent growth inhibition than standard-of-care platinum-based chemotherapy and caused reduced mesenchymal characteristics in patient-derived xenografts. An important consideration is the nature of our patient-derived cultures being treatment-naïve (pre NACT). Even prior to the current advent of the Keynote 522 regimen of Pembrolizumab in addition to TAC [51], the TAC regimen provided important clinical benefits. In particular, chemo-naïve TNBCs in the clinic are initially highly responsive to doxorubicin, and this was reflected in the strong responses seen amongst the patient-derived cultures. It is possible that chemosensitisation was not seen in the TNBC cultures because they are already exquisitely sensitive to doxorubicin. Further studies will seek to treat the cells initially with doxorubicin and then treat with eribulin versus vehicle after the onset of doxorubicin resistance, followed by re-challenge.

On the other hand, there is potential for EMT to be associated with resistance to HER2-targeted therapies, which may be reflected in the strong partial or E/M-hybrid phenotype present in the HER2+ CBCa50 cells and chemosensitisation seen with both CBCa50, and minimally in the triple positive sample (ER+/PR+/HER2+) CBCa51. Little has been reported on EMT in HER2-positive breast cancer, however EMT has been seen in trastuzumab resistance [52,53,54]. Furthermore, HER2 has also been shown to induce TGFβ signalling and subsequently promote EMT, which may explain the eribulin-induced chemosensitisation and epithelial reprograming observed in the study [55]. Regarding eribulin response, in the SOLTI NeoEribulin trial which was conducted in HER2-negative breast cancers, patients with a HER2-enriched molecular profile appeared to benefit the most from eribulin [28]. Further work on HER2+ breast cancer cultures may provide some explanation.

## 5. Conclusions

In summary, our findings indicate that eribulin can induce clinically relevant phenotypic changes by promoting the acquisition of epithelial features and reducing mesenchymal characteristics across breast cancer patient samples and cell lines, which may limit metastatic dissemination and create a tumour state more sensitive to subsequent therapies. The capacity for tumour cells to respond to eribulin appears to be dependent on their epithelial–mesenchymal status and breast cancer subtype, with more evident effects in HER2-positive and hormone-receptor-positive patient-derived cultures in the present study. These findings support the clinical relevance of incorporating EMT biomarkers in tumour profiling to identify patients who can benefit from eribulin and other treatments that target the EMT/MET processes.

## Figures and Tables

**Table 1:** Patient-derived cultures, clinical subtype and prior treatment status.

Case ID	Clinical Subtype	Prior Treatment
CBCa23	ER−/PR−/HER2−	None
CBCa41	ER−/PR−/HER2−	None
CBCa42	ER−/PR−/HER2−	None
CBCa50	ER−/PR−/HER2+	None
CBCa51	ER+/PR+/HER2+	None

**Table 2:** Clinical characteristics of breast cancer cohort receiving standard chemotherapy.

		N	(%) *
Total		30	
Age			
	<50	19	(68)
	>50	9	(32)
	Unknown	2	-
Histological subtype			
	Invasive ductal	23	(82)
	Invasive lobular	5	(18)
	Unknown	2	-
Histological Grade			
	Grade 1	3	(11)
	Grade 2	9	(32)
	Grade 3	16	(57)
	Unknown	2	-
Hormone receptor			
	Positive	15	(50)
	Negative	15	(50)
HER2 Status			
	Positive	9	(30)
	Negative	21	(70)
Lymph vascular invasion			
	Present	10	(45)
	Absent	12	(55)
	Unknown	8	-
Lymph Node Involvement			
	Positive	13	(46)
	Negative	15	(54)
	Unknown	2	-

* Calculated from total with known status for variable.

## Data Availability

The data associated with this study is included within both the manuscript and its Supplementary Materials or was uploaded during submission. Any additional data is available from the corresponding author upon reasonable request.

## Supplementary Materials

The following supporting information can be downloaded at https://www.mdpi.com/article/10.3390/cancers18040598/s1, Figure S1: Evidence of Estrogen Receptor and Proliferative Marker Ki67 Expression in CBCa51 Triple-Positive Breast Cancer Primary Cell Culture; Figure S2: Statistical Comparison of Primary Patient-Derived Cell Responses to Eribulin Treatment Denoted by Subtype; Table S1: Statistical Summary of Nanostring Gene Expression Analysis; Table S2: A comparison of overall survival and progression-free survival (disease control) benefits for a range of therapeutic agents in randomized clinical trials according to their impact on EMT [20,30,45,56,57,58,59,60,61,62,63,64,65,66,67,68,69,70,71,72,73,74,75,76,77,78,79,80,81,82,83,84].

## References

[1] Kalluri R., Weinberg R.A. (2009). The basics of epithelial-mesenchymal transition. J. Clin. Investig..

[2] Thompson E.W., Redfern A.D., Brabletz S., Berx G., Agarwal V., Ganesh K., Huang R.Y., Ishay-Ronen D., Savagner P., Sheng G. (2025). EMT and cancer: What clinicians should know. Nat. Rev. Clin. Oncol..

[3] Sahoo S., Nayak S.P., Hari K., Purkait P., Mandal S., Kishore A., Levine H., Jolly M.K. (2021). Immunosuppressive Traits of the Hybrid Epithelial/Mesenchymal Phenotype. Front. Immunol..

[4] Wang L., Saci A., Szabo P.M., Chasalow S.D., Castillo-Martin M., Domingo-Domenech J., Siefker-Radtke A., Sharma P., Sfakianos J.P., Gong Y. (2018). EMT- and stroma-related gene expression and resistance to PD-1 blockade in urothelial cancer. Nat. Commun..

[5] Yao J., Caballero O.L., Huang Y., Lin C., Rimoldi D., Behren A., Cebon J.S., Hung M.C., Weinstein J.N., Strausberg R.L. (2016). Altered Expression and Splicing of ESRP1 in Malignant Melanoma Correlates with Epithelial-Mesenchymal Status and Tumor-Associated Immune Cytolytic Activity. Cancer Immunol. Res..

[6] Schmid P., Adams S., Rugo H.S., Schneeweiss A., Barrios C.H., Iwata H., Diéras V., Hegg R., Im S.-A., Shaw Wright G. (2018). Atezolizumab and Nab-Paclitaxel in Advanced Triple-Negative Breast Cancer. N. Engl. J. Med..

[7] Goto W., Kashiwagi S., Asano Y., Takada K., Morisaki T., Fujita H., Takashima T., Ohsawa M., Hirakawa K., Ohira M. (2018). Eribulin Promotes Antitumor Immune Responses in Patients with Locally Advanced or Metastatic Breast Cancer. Anticancer Res..

[8] Redfern A., Agarwal V., Thompson E.W. (2019). Hypoxia as a signal for prison breakout in cancer. Curr. Opin. Clin. Nutr. Metab. Care.

[9] Valachis A., Polyzos N.P., Patsopoulos N.A., Georgoulias V., Mavroudis D., Mauri D. (2010). Bevacizumab in metastatic breast cancer: A meta-analysis of randomized controlled trials. Breast Cancer Res. Treat..

[10] Xu H., Rahimpour S., Nesvick C.L., Zhang X., Ma J., Zhang M., Zhang G., Wang L., Yang C., Hong C.S. (2015). Activation of hypoxia signaling induces phenotypic transformation of glioma cells: Implications for bevacizumab antiangiogenic therapy. Oncotarget.

[11] Ito K., Hamamichi S., Abe T., Akagi T., Shirota H., Kawano S., Asano M., Asano O., Yokoi A., Matsui J. (2017). Antitumor effects of eribulin depend on modulation of the tumor microenvironment by vascular remodeling in mouse models. Cancer Sci..

[12] He B., Wood K.H., Li Z.J., Ermer J.A., Li J., Bastow E.R., Sakaram S., Darcy P.K., Spalding L.J., Redfern C.T. (2025). Selective tubulin-binding drugs induce pericyte phenotype switching and anti-cancer immunity. EMBO Mol. Med..

[13] Chen Y., McAndrews K.M., Kalluri R. (2021). Clinical and therapeutic relevance of cancer-associated fibroblasts. Nat. Rev. Clin. Oncol..

[14] Fiori M.E., Di Franco S., Villanova L., Bianca P., Stassi G., De Maria R. (2019). Cancer-associated fibroblasts as abettors of tumor progression at the crossroads of EMT and therapy resistance. Mol. Cancer.

[15] Luong T., Cukierman E. (2022). Eribulin normalizes pancreatic cancer-associated fibroblasts by simulating selected features of TGFβ inhibition. BMC Cancer.

[16] Towle M.J., Nomoto K., Asano M., Kishi Y., Yu M.J., Littlefield B.A. (2012). Broad spectrum preclinical antitumor activity of eribulin (Halaven(R)): Optimal effectiveness under intermittent dosing conditions. Anticancer Res..

[17] Towle M.J., Salvato K.A., Wels B.F., Aalfs K.K., Zheng W., Seletsky B.M., Zhu X., Lewis B.M., Kishi Y., Yu M.J. (2011). Eribulin induces irreversible mitotic blockade: Implications of cell-based pharmacodynamics for in vivo efficacy under intermittent dosing conditions. Cancer Res..

[18] Yang J., Antin P., Berx G., Blanpain C., Brabletz T., Bronner M., Campbell K., Cano A., Casanova J., Christofori G. (2020). Guidelines and definitions for research on epithelial-mesenchymal transition. Nat. Rev. Mol. Cell Biol..

[19] Yoshida T., Ozawa Y., Kimura T., Sato Y., Kuznetsov G., Xu S., Uesugi M., Agoulnik S., Taylor N., Funahashi Y. (2014). Eribulin mesilate suppresses experimental metastasis of breast cancer cells by reversing phenotype from epithelial-mesenchymal transition (EMT) to mesenchymal-epithelial transition (MET) states. Br. J. Cancer.

[20] Terashima M., Sakai K., Togashi Y., Hayashi H., De Velasco M.A., Tsurutani J., Nishio K. (2014). Synergistic antitumor effects of S-1 with eribulin in vitro and in vivo for triple-negative breast cancer cell lines. Springerplus.

[21] Kitahara H., Hirai M., Kato K., Bou-Gharios G., Nakamura H., Kawashiri S. (2016). Eribulin sensitizes oral squamous cell carcinoma cells to cetuximab via induction of mesenchymal-to-epithelial transition. Oncol. Rep..

[22] Li Q., Hopcraft S.E., Lange P.T., Pluta L., Dittmer D.P., Moody C.A., Damania B. (2025). KSHV and HPV modulate epithelial-to-mesenchymal transition in oral epithelial cells. mBio.

[23] Hayashi T., Kobayashi N., Ushida K., Asai N., Nakano S., Fujii K., Ando T., Utsumi T. (2023). Effect of eribulin on epithelial-mesenchymal transition plasticity in metastatic breast cancer: An exploratory, prospective study. Genes Cells.

[24] Redfern A.D., Spalding L.J., Thompson E.W. (2018). The Kraken Wakes: Induced EMT as a driver of tumour aggression and poor outcome. Clin. Exp. Metastasis.

[25] Twelves C., Cortes J., Kaufman P.A., Yelle L., Awada A., Binder T.A., Olivo M., Song J., O’Shaughnessy J.A., Jove M. (2015). “New” metastases are associated with a poorer prognosis than growth of pre-existing metastases in patients with metastatic breast cancer treated with chemotherapy. Breast Cancer Res..

[26] Yamamoto M., Sakane K., Tominaga K., Gotoh N., Niwa T., Kikuchi Y., Tada K., Goshima N., Semba K., Inoue J.I. (2017). Intratumoral bidirectional transitions between epithelial and mesenchymal cells in triple-negative breast cancer. Cancer Sci..

[27] Bock N., Forouz F., Hipwood L., Clegg J., Jeffery P., Gough M., van Wyngaard T., Pyke C., Adams M.N., Bray L.J. (2023). GelMA, Click-Chemistry Gelatin and Bioprinted Polyethylene Glycol-Based Hydrogels as 3D Ex Vivo Drug Testing Platforms for Patient-Derived Breast Cancer Organoids. Pharmaceutics.

[28] Pascual T., Oliveira M., Villagrasa P., Ortega V., Paré L., Bermejo B., Morales S., Amillano K., López R., Galván P. (2021). Neoadjuvant eribulin in HER2-negative early-stage breast cancer (SOLTI-1007-NeoEribulin): A multicenter, two-cohort, non-randomized phase II trial. NPJ Breast Cancer.

[29] Redfern A., McLaren S., Dissanayake V., Chan A., Zeps N., Dobrovic A., Soon L., Thompson E., Christobel S. (2016). Abstract P1-05-03: Predictive value of de novo and induced epithelial-mesenchymal transition in locally advanced breast cancer treated with neoadjuvant chemotherapy. Cancer Res..

[30] Cursons J., Leuchowius K.J., Waltham M., Tomaskovic-Crook E., Foroutan M., Bracken C.P., Redfern A., Crampin E.J., Street I., Davis M.J. (2015). Stimulus-dependent differences in signalling regulate epithelial-mesenchymal plasticity and change the effects of drugs in breast cancer cell lines. Cell Commun. Signal. CCS.

[31] Carr R.M., Qiao G., Qin J., Jayaraman S., Prabhakar B.S., Maker A.V. (2016). Targeting the metabolic pathway of human colon cancer overcomes resistance to TRAIL-induced apoptosis. Cell Death Discov..

[32] Wahi K., Freidman N., Wang Q., Devadason M., Quek L.-E., Pang A., Lloyd L., Larance M., Zanini F., Harvey K. (2024). Macropinocytosis mediates resistance to loss of glutamine transport in triple-negative breast cancer. EMBO J..

[33] Stringer C., Wang T., Michaelos M., Pachitariu M. (2021). Cellpose: A generalist algorithm for cellular segmentation. Nat. Methods.

[34] van der Walt S., Schönberger J.L., Nunez-Iglesias J., Boulogne F., Warner J.D., Yager N., Gouillart E., Yu T. (2014). scikit-image: Image processing in Python. PeerJ.

[35] Traag V.A., Waltman L., van Eck N.J. (2019). From Louvain to Leiden: Guaranteeing well-connected communities. Sci. Rep..

[36] Hunter J.D. (2007). Matplotlib: A 2D Graphics Environment. Comput. Sci. Eng..

[37] Waskom M. (2021). seaborn: Statistical data visualization. J. Open Source Softw..

[38] Bagheri M., Mohamed G.A., Mohamed Saleem M.A., Ognjenovic N.B., Lu H., Kolling F.W., Wilkins O.M., Das S., LaCroix I.S., Nagaraj S.H. (2024). Pharmacological induction of chromatin remodeling drives chemosensitization in triple-negative breast cancer. Cell Rep. Med..

[39] Kröger C., Afeyan A., Mraz J., Eaton E.N., Reinhardt F., Khodor Y.L., Thiru P., Bierie B., Ye X., Burge C.B. (2019). Acquisition of a hybrid E/M state is essential for tumorigenicity of basal breast cancer cells. Proc. Natl. Acad. Sci. USA.

[40] Bierie B., Pierce S.E., Kroeger C., Stover D.G., Pattabiraman D.R., Thiru P., Liu Donaher J., Reinhardt F., Chaffer C.L., Keckesova Z. (2017). Integrin-β4 identifies cancer stem cell-enriched populations of partially mesenchymal carcinoma cells. Proc. Natl. Acad. Sci. USA.

[41] Martínez-Cenalmor P., Martínez A.E., Moneo-Corcuera D., González-Jiménez P., Pérez-Sala D. (2024). Oxidative stress elicits the remodeling of vimentin filaments into biomolecular condensates. Redox Biol..

[42] Pérez-Sala D., Zorrilla S. (2025). Versatility of vimentin assemblies: From filaments to biomolecular condensates and back. Eur. J. Cell Biol..

[43] Guo M., Wong I.Y., Moore A.S., Medalia O., Lippincott-Schwartz J., Weitz D.A., Goldman R.D. (2025). Vimentin intermediate filaments as structural and mechanical coordinators of mesenchymal cells. Nat. Cell Biol..

[44] Demetri G.D., Schöffski P., Grignani G., Blay J.Y., Maki R.G., Van Tine B.A., Alcindor T., Jones R.L., D’Adamo D.R., Guo M. (2017). Activity of Eribulin in Patients with Advanced Liposarcoma Demonstrated in a Subgroup Analysis from a Randomized Phase III Study of Eribulin Versus Dacarbazine. J. Clin. Oncol..

[45] Twelves C., Cortes J., Vahdat L., Olivo M., He Y., Kaufman P.A., Awada A. (2014). Efficacy of eribulin in women with metastatic breast cancer: A pooled analysis of two phase 3 studies. Breast Cancer Res. Treat..

[46] Hara Y., Nakanishi Y., Hirotani Y., Enomoto K., Masuda S., Tada K. (2025). Predicting the Efficacy of Eribulin in Metastatic Breast Cancer by Assessing E-Cadherin and Vimentin Expression. Acta Histochem. Cytochem..

[47] Chan A., Gill J., Chih H., Wright S.C.E., Vasilevski N., Eichhorn P.J.A. (2024). Influence of Epithelial-Mesenchymal Transition on Risk of Relapse and Outcome to Eribulin or Cyclin-Dependent Kinase Inhibitors in Metastatic Breast Cancer. JCO Precis. Oncol..

[48] Funahashi Y., Okamoto K., Adachi Y., Semba T., Uesugi M., Ozawa Y., Tohyama O., Uehara T., Kimura T., Watanabe H. (2014). Eribulin mesylate reduces tumor microenvironment abnormality by vascular remodeling in preclinical human breast cancer models. Cancer Sci..

[49] Jeong Y.G., Katuwal N.B., Kang M.S., Ghosh M., Hong S.D., Park S.M., Kim S.G., Kim T.H., Moon Y.W. (2023). Combined PI3K Inhibitor and Eribulin Enhances Anti-Tumor Activity in Preclinical Models of Paclitaxel-Resistant, PIK3CA-Mutated Endometrial Cancer. Cancers.

[50] Ho G.Y., Kyran E.L., Bedo J., Wakefield M.J., Ennis D.P., Mirza H.B., Vandenberg C.J., Lieschke E., Farrell A., Hadla A. (2022). Epithelial-to-Mesenchymal Transition Supports Ovarian Carcinosarcoma Tumorigenesis and Confers Sensitivity to Microtubule Targeting with Eribulin. Cancer Res..

[51] Schmid P., Cortes J., Dent R., McArthur H., Pusztai L., Kümmel S., Denkert C., Park Y.H., Hui R., Harbeck N. (2024). Overall Survival with Pembrolizumab in Early-Stage Triple-Negative Breast Cancer. N. Engl. J. Med..

[52] Oliveras-Ferraros C., Corominas-Faja B., Cufí S., Vazquez-Martin A., Martin-Castillo B., Iglesias J.M., López-Bonet E., Martin Á.G., Menendez J.A. (2012). Epithelial-to-mesenchymal transition (EMT) confers primary resistance to trastuzumab (Herceptin). Cell Cycle.

[53] Ye X., Liu Q., Qin X., Ma Y., Sheng Q., Wu X., Chen S., Huang L., Sun Y. (2024). BCAR4 facilitates trastuzumab resistance and EMT in breast cancer via sponging miR-665 and interacting with YAP1. FASEB J..

[54] Burnett J.P., Korkaya H., Ouzounova M.D., Jiang H., Conley S.J., Newman B.W., Sun L., Connarn J.N., Chen C.S., Zhang N. (2015). Trastuzumab resistance induces EMT to transform HER2(+) PTEN(−) to a triple negative breast cancer that requires unique treatment options. Sci. Rep..

[55] Gupta P., Srivastava S.K. (2014). HER2 mediated de novo production of TGFβ leads to SNAIL driven epithelial-to-mesenchymal transition and metastasis of breast cancer. Mol. Oncol..

[56] Saxena M., Stephens M.A., Pathak H., Rangarajan A. (2011). Transcription factors that mediate epithelial-mesenchymal transition lead to multidrug resistance by upregulating ABC transporters. Cell Death Dis..

[57] Tannock I.F., Osoba D., Stockler M.R., Ernst D.S., Neville A.J., Moore M.J., Armitage G.R., Wilson J.J., Venner P.M., Coppin C.M. (1996). Chemotherapy with mitoxantrone plus prednisone or prednisone alone for symptomatic hormone-resistant prostate cancer: A Canadian randomized trial with palliative end points. J. Clin. Oncol..

[58] Miyazaki S., Kikuchi H., Iino I., Uehara T., Setoguchi T., Fujita T., Hiramatsu Y., Ohta M., Kamiya K., Kitagawa K. (2014). Anti-VEGF antibody therapy induces tumor hypoxia and stanniocalcin 2 expression and potentiates growth of human colon cancer xenografts. Int. J. Cancer.

[59] Creighton C.J., Li X., Landis M., Dixon J.M., Neumeister V.M., Sjolund A., Rimm D.L., Wong H., Rodriguez A., Herschkowitz J.I. (2009). Residual breast cancers after conventional therapy display mesenchymal as well as tumor-initiating features. Proc. Natl. Acad. Sci. USA.

[60] Martín M., Loibl S., von Minckwitz G., Morales S., Martinez N., Guerrero A., Anton A., Aktas B., Schoenegg W., Muñoz M. (2015). Phase III trial evaluating the addition of bevacizumab to endocrine therapy as first-line treatment for advanced breast cancer: The letrozole/fulvestrant and avastin (LEA) study. J. Clin. Oncol..

[61] Zhang G., Tian X., Li Y., Wang Z., Li X., Zhu C. (2018). miR-27b and miR-34a enhance docetaxel sensitivity of prostate cancer cells through inhibiting epithelial-to-mesenchymal transition by targeting ZEB1. Biomed. Pharmacother..

[62] Marín-Aguilera M., Codony-Servat J., Reig Ò., Lozano J.J., Fernández P.L., Pereira M.V., Jiménez N., Donovan M., Puig P., Mengual L. (2014). Epithelial-to-mesenchymal transition mediates docetaxel resistance and high risk of relapse in prostate cancer. Mol. Cancer Ther..

[63] Li C., Xiang A., Chen X., Yin K., Lu J., Yin W. (2017). Optimizing the treatment of bevacizumab as first-line therapy for human epidermal growth factor receptor 2 (HER2)-negative advanced breast cancer: An updated meta-analysis of published randomized trials. OncoTargets Ther..

[64] Jayachandran A., Anaka M., Prithviraj P., Hudson C., McKeown S.J., Lo P.-H., Vella L.J., Goding C.R., Cebon J., Behren A. (2014). Thrombospondin 1 promotes an aggressive phenotype through epithelial-to-mesenchymal transition in human melanoma. Oncotarget.

[65] McArthur G.A., Chapman P.B., Robert C., Larkin J., Haanen J.B., Dummer R., Ribas A., Hogg D., Hamid O., Ascierto P.A. (2014). Safety and efficacy of vemurafenib in BRAF(V600E) and BRAF(V600K) mutation-positive melanoma (BRIM-3): Extended follow-up of a phase 3, randomised, open-label study. Lancet Oncol..

[66] Mikami S., Mizuno R., Kosaka T., Saya H., Oya M., Okada Y. (2015). Expression of TNF-α and CD44 is implicated in poor prognosis, cancer cell invasion, metastasis and resistance to the sunitinib treatment in clear cell renal cell carcinomas. Int. J. Cancer.

[67] Motzer R.J., Hutson T.E., Tomczak P., Michaelson M.D., Bukowski R.M., Oudard S., Negrier S., Szczylik C., Pili R., Bjarnason G.A. (2009). Overall survival and updated results for sunitinib compared with interferon alfa in patients with metastatic renal cell carcinoma. J. Clin. Oncol..

[68] Yardley D.A., Noguchi S., Pritchard K.I., Burris H.A., Baselga J., Gnant M., Hortobagyi G.N., Campone M., Pistilli B., Piccart M. (2013). Everolimus plus exemestane in postmenopausal patients with HR(+) breast cancer: BOLERO-2 final progression-free survival analysis. Adv. Ther..

[69] Piccart M., Hortobagyi G.N., Campone M., Pritchard K.I., Lebrun F., Ito Y., Noguchi S., Perez A., Rugo H.S., Deleu I. (2014). Everolimus plus exemestane for hormone-receptor-positive, human epidermal growth factor receptor-2-negative advanced breast cancer: Overall survival results from BOLERO-2. Ann. Oncol..

[70] Nagai T., Arao T., Furuta K., Sakai K., Kudo K., Kaneda H., Tamura D., Aomatsu K., Kimura H., Fujita Y. (2011). Sorafenib inhibits the hepatocyte growth factor-mediated epithelial mesenchymal transition in hepatocellular carcinoma. Mol. Cancer Ther..

[71] Llovet J.M., Ricci S., Mazzaferro V., Hilgard P., Gane E., Blanc J.F., de Oliveira A.C., Santoro A., Raoul J.L., Forner A. (2008). Sorafenib in advanced hepatocellular carcinoma. N. Engl. J. Med..

[72] Shen T., Cheng X., Xia C., Li Q., Gao Y., Pan D., Zhang X., Zhang C., Li Y. (2019). Erlotinib inhibits colon cancer metastasis through inactivation of TrkB-dependent ERK signaling pathway. J. Cell. Biochem..

[73] Wu Y.L., Lee J.S., Thongprasert S., Yu C.J., Zhang L., Ladrera G., Srimuninnimit V., Sriuranpong V., Sandoval-Tan J., Zhu Y. (2013). Intercalated combination of chemotherapy and erlotinib for patients with advanced stage non-small-cell lung cancer (FASTACT-2): A randomised, double-blind trial. Lancet Oncol..

[74] Lamouille S., Derynck R. (2011). Emergence of the phosphoinositide 3-kinase-Akt-mammalian target of rapamycin axis in transforming growth factor-β-induced epithelial-mesenchymal transition. Cells Tissues Organs.

[75] André F., Ciruelos E.M., Juric D., Loibl S., Campone M., Mayer I.A., Rubovszky G., Yamashita T., Kaufman B., Lu Y.S. (2021). Alpelisib plus fulvestrant for PIK3CA-mutated, hormone receptor-positive, human epidermal growth factor receptor-2-negative advanced breast cancer: Final overall survival results from SOLAR-1. Ann. Oncol..

[76] Boz Er A.B., Er I. (2024). Targeting ITGβ3 to Overcome Trastuzumab Resistance through Epithelial-Mesenchymal Transition Regulation in HER2-Positive Breast Cancer. Int. J. Mol. Sci..

[77] Marty M., Cognetti F., Maraninchi D., Snyder R., Mauriac L., Tubiana-Hulin M., Chan S., Grimes D., Antón A., Lluch A. (2005). Randomized phase II trial of the efficacy and safety of trastuzumab combined with docetaxel in patients with human epidermal growth factor receptor 2-positive metastatic breast cancer administered as first-line treatment: The M77001 study group. J. Clin. Oncol..

[78] Martin S.K., Pu H., Penticuff J.C., Cao Z., Horbinski C., Kyprianou N. (2016). Multinucleation and Mesenchymal-to-Epithelial-Transition Alleviate Resistance to Combined Cabazitaxel and Antiandrogen Therapy in Advanced Prostate Cancer. Cancer Res..

[79] de Bono J., Oudard S., Ozguroglu M., Hansen S., Machiels J.-P., Kocak I., Gravis G., Bodrogi I., Mackenzie M., Shen L. (2010). Prednisone plus cabazitaxel or mitoxantrone for metastatic castration-resistant prostate cancer progressing after docetaxel treatment: A randomised open-label trial. Lancet.

[80] Geng W., Thomas H., Chen Z., Yan Z., Zhang P., Zhang M., Huang W., Ren X., Wang Z., Ding K. (2024). Mechanisms of acquired resistance to HER2-Positive breast cancer therapies induced by HER3: A comprehensive review. Eur. J. Pharmacol..

[81] Swain S.M., Miles D., Kim S.B., Im Y.H., Im S.A., Semiglazov V., Ciruelos E., Schneeweiss A., Loi S., Monturus E. (2020). Pertuzumab, trastuzumab, and docetaxel for HER2-positive metastatic breast cancer (CLEOPATRA): End-of-study results from a double-blind, randomised, placebo-controlled, phase 3 study. Lancet Oncol..

[82] Shah P., Gau Y., Sabnis G. (2014). Histone deacetylase inhibitor entinostat reverses epithelial to mesenchymal transition of breast cancer cells by reversing the repression of E-cadherin. Breast Cancer Res. Treat..

[83] Yardley D.A., Ismail-Khan R.R., Melichar B., Lichinitser M., Munster P.N., Klein P.M., Cruickshank S., Miller K.D., Lee M.J., Trepel J.B. (2013). Randomized Phase II, Double-Blind, Placebo-Controlled Study of Exemestane With or Without Entinostat in Postmenopausal Women With Locally Recurrent or Metastatic Estrogen Receptor-Positive Breast Cancer Progressing on Treatment With a Nonsteroidal Aromatase Inhibitor. J. Clin. Oncol..

[84] Kawano S., Asano M., Adachi Y., Matsui J. (2016). Antimitotic and Non-Mitotic Effects of Eribulin Mesilate in Soft Tissue Sarcoma. Anticancer Res..

