# Supplementary material for: Effects of Eribulin on Epithelial–Mesenchymal Plasticity in Patient-Derived Breast Cancer Cultures and Excised Tissues
Source: Cancers (Basel). 2026 Feb 11;18(4):598. doi: 10.3390/cancers18040598 (PMC12939509; doi:10.3390/cancers18040598)
Supplement: Supplementary file 1 [file cancers-18-00598-s001.zip › cancers-4118142-supplementary.pdf]

Table S1. – Statistical Summary of Nanostring Gene Expression Analysis. Gene fold change values are reported following TAC treatment (TAC FC) versus Eribulin (ERI FC) along with statistical summary of the two-stage linear step-up procedure of Benjamini, Krieger and Yekutieli. SE – standard error, df – degrees of freedom.

Gene	Discovery	P value	TAC FC	ERI FC	Difference	SE	t ratio	df	q value
AR	No	0.171818492	0.2415	-0.1036	0.3452	0.2515	1.372	162	0.205293474
CAMK2N1	Yes	7.47382E-05	0.9164	0.02824	0.8882	0.2185	4.065	162	0.000299664
CAV1	Yes	2.70308E-06	1.731	0.4023	1.329	0.2732	4.864	162	1.56103E-05
CCND1	No	0.999263057	-0.06052	-0.06032	-0.0002	0.216	0.000925	162	0.692489298
CD24	No	0.097568155	-0.3861	0.09057	-0.4766	0.286	1.666	162	0.130028329
CD44	Yes	0.002139686	0.5882	0.1193	0.4689	0.1503	3.12	162	0.00674001
CDH1	Yes	0.030093438	-0.4745	0.1027	-0.5772	0.2638	2.188	162	0.047397165
CDKN1A	Yes	0.000146056	1.497	0.5676	0.929	0.2388	3.89	162	0.000506085
CDKN3	No	0.662513794	-0.455	-0.3536	-0.1014	0.2319	0.4372	162	0.531146473
CLDN4	No	0.084634816	-0.247	0.2443	-0.4913	0.2832	1.735	162	0.120681107
CLDN7	No	0.214999074	-0.5902	-0.2871	-0.3032	0.2435	1.245	162	0.24832393
CTNNB1	Yes	0.019089177	0.3677	0.1033	0.2644	0.1117	2.367	162	0.036746665
CXCL14	No	0.692833167	0.6988	0.5484	0.1504	0.3802	0.3957	162	0.531146473
EGFR	Yes	0.025812196	0.9375	0.3691	0.5684	0.2526	2.25	162	0.04471963
EMP3	Yes	4.33132E-05	0.7926	-0.05285	0.8455	0.2011	4.204	162	0.0002144
ESR1	No	0.353104819	0.2092	0.5285	-0.3192	0.3428	0.9313	162	0.324115602
ESRP1	No	0.419501871	-0.2505	-0.08204	-0.1684	0.2081	0.8094	160	0.363393496
EZH2	No	0.705129516	-0.4275	-0.3528	-0.07475	0.1972	0.3791	162	0.531146473
FOXA1	No	0.550487127	0.141	0.3602	-0.2191	0.3663	0.5983	159	0.465228755
GREM1	No	0.087071506	0.462	-0.04386	0.5058	0.2938	1.721	162	0.120681107
GRHL2	No	0.287525035	-0.3055	-0.08631	-0.2192	0.2054	1.067	162	0.290624795
GUSB	Yes	0.023768647	0.3696	0.03111	0.3385	0.1483	2.282	162	0.043346507
ID4	Yes	0.003065057	1.635	0.5891	1.046	0.3479	3.006	162	0.008169555
INHBA	No	0.074205399	0.3594	-0.1312	0.4905	0.273	1.797	162	0.111792047
KRT14	No	0.327221009	0.4413	1.066	-0.6251	0.636	0.9829	154	0.314950222
KRT17	No	0.829417536	0.8323	0.9506	-0.1182	0.5479	0.2158	159	0.586516686
KRT5	No	0.374698907	0.3198	0.7664	-0.4466	0.5016	0.8902	159	0.332905567
KRT6A	No	0.794574917	-0.2327	-0.09569	-0.137	0.5251	0.2609	141	0.582959611
KRT6B	No	0.252766528	-0.007589	0.5283	-0.5359	0.4668	1.148	153	0.273698756
LAMC2	No	0.293560399	0.2725	-0.02825	0.3008	0.2854	1.054	159	0.290624795
MAP2K1	Yes	2.21205E-07	0.5231	0.08399	0.4391	0.08114	5.412	162	2.55492E-06
MKI67	No	0.60893335	-0.4109	-0.5521	0.1412	0.2755	0.5126	162	0.502370014
MMP11	No	0.234272489	-1.018	-0.589	-0.4294	0.3597	1.194	162	0.261856186
MYC	Yes	0.028156037	1.039	0.534	0.5049	0.2279	2.215	162	0.046457461
NF1	Yes	0.009335278	0.3011	0.04364	0.2575	0.09785	2.631	162	0.019027493
PGR	No	0.678539899	0.01865	0.172	-0.1534	0.3694	0.4152	158	0.531146473
PRC1	No	0.807563097	-0.3859	-0.3266	-0.05926	0.2429	0.244	161	0.582959611
RRM2	No	0.355451454	-0.9038	-0.597	-0.3068	0.3311	0.9267	162	0.324115602
S100A8	Yes	0.008168471	0.9873	0.02681	0.9605	0.3586	2.678	162	0.017689845
S100A9	Yes	0.002807247	1.001	-0.08144	1.083	0.3567	3.035	162	0.008105924
SCGB2A2	No	0.147607398	-0.7053	0.4696	-1.175	0.8072	1.455	151	0.182664155
SNAI1	Yes	0.004560781	0.5884	0.01117	0.5772	0.2007	2.877	162	0.011287934
SPINT2	No	0.112887856	-0.3338	-0.06307	-0.2707	0.1698	1.594	162	0.144872748
TGFBR2	Yes	8.51742E-08	1.21	0.09048	1.12	0.1996	5.611	162	1.47564E-06
TK1	No	0.283765511	-0.1871	-0.432	0.2449	0.2277	1.076	158	0.290624795
TWIST1	Yes	7.78349E-05	1.919	0.6178	1.301	0.3208	4.055	162	0.000299664
TWIST2	Yes	0.005230535	1.249	0.439	0.8097	0.286	2.831	162	0.012082536
VIM	Yes	8.79265E-07	1.155	0.1723	0.9825	0.1921	5.115	162	6.09331E-06
ZEB1	Yes	3.25876E-08	1.245	0.1408	1.105	0.1902	5.808	162	1.12916E-06
ZEB2	Yes	3.07028E-07	0.9308	-0.2687	1.2	0.2245	5.342	162	2.65963E-06

Table S2. - A comparison of overall survival and progression-free survival (disease control) benefits for a range of therapeutic agents in randomized clinical trials according to their impact on EMT. A ratio below 1.0 indicates that the overall survival benefit is shorter than the increase in disease control period implying accelerated cancer progression after treatment completion. A ratio above 1.0 indicates extended survival beyond that of disease control implying an inhibition of cancer progression after treatment completion. Citation numbers included link back to the reference list of the main document.

Disease	Treatment	Preclinical Evidence	Process	Progression-Free Survival Benefit	Overall Survival Benefit	OS-PFS Benefit Ratio	Pre-clinical Reference	Clinical Reference	Clinical Cohort Size(s)
Prostate Cancer	Mitoxantrone	Anthracyclines induce EMT and drug resistance in a range of tumour types	EMT	5.7	0.2	**0.04**	[56]	[57]	161
Breast cancer	Bevacizumab & Letrozole	Neoadjuvant letrozole induces EMT in breast cancer bevacizumab induces EMT in cecal & glial tumours	EMT	4.9	0.3	**0.06**	[58,59]	[60]	1174
Breast cancer	Bevacizumab & Taxane	Paclitaxel drives EMT in breast cancer cells bevacizumab induces EMT in cecal & glial tumours	EMT	5.9	1.5	**0.25**	[58,61,62]	[63]	3,984
Melanoma	Vemurafenib	Vemurafanib resistance was accompanied by elevated THBSG1 levels that led to EMT	EMT	5.3	3.9	**0.74**	[64]	[65]	675
Renal carcinoma	Sunitinib	TNF-α drives ccRCC progression by inducing EMT & CD44 inducing sunitinib resistance	EMT	6	4.6	**0.77**	[66]	[67]	750
Breast Cancer	Everolimus	Everolimus is cytotoxic to cells mesenchymally transitioned due to EGFR exposure	MET	4.6	4.4	**0.96**	[30]	[68,69]	724
liver cancer	Sorafenib	Sorafenib downregulates SNAIL by inhibiting MAPK signaling, thereby inhibiting the EMT in HCC cells	MET	2.7	2.8	**1.04**	[70]	[71]	602
Non-small cell lung cancer	Erlotinib	Erlotinib inhibits colon cancer cell EMT, migration, and invasion by inactivating ERK signaling pathway	MET	9.9	10.8	**1.09**	[72]	[73]	451
Breast Cancer	Alpelisib	PI3 kinase is a core EMT activating pathway	MET	5.7	7.9	**1.49**	[74]	[75]	572
Breast Cancer	Trastuzumab	Trastuzumab initially downregulates EMT-TFs TWIST2, Snail, Zeb1. With sustained blockade EMT reappears driven by PI3K	MET	5.6	8.5	**1.52**	[76]	[77]	186
Prostate Cancer	Carbazitaxel	Combination therapy of human CRPC xenografts with carbazitaxel and enzalutamide reversed EMT	MET	1.4	2.4	**1.71**	[78]	[79]	755

Breast Cancer	Pertuzumab	By blocking HER2-HER3 dimer signaling pertuzumab blocks PI3K activation, a source of EMT in HER2 targeting resistance	MET	6.3	16.3	**2.59**	[74,80]	[81]	1196
Breast Cancer	Eribulin	Eribulin reverses EMT in breast cancer cells and breast cancer xenografts	MET	0.6	2.4	**4.00**	[20]	[45]	1,864
Breast cancer	Entinostat	Entinostat reverses epithelial to mesenchymal transition of breast cancer cells	MET	2	8.3	**4.20**	[82]	[83]	130
Liposarcoma	Eribulin	Eribulin promotes differentiation of liposarcoma and leiomyosarcoma	MET	1.2	7.2	**6.00**	[84]	[44]	143

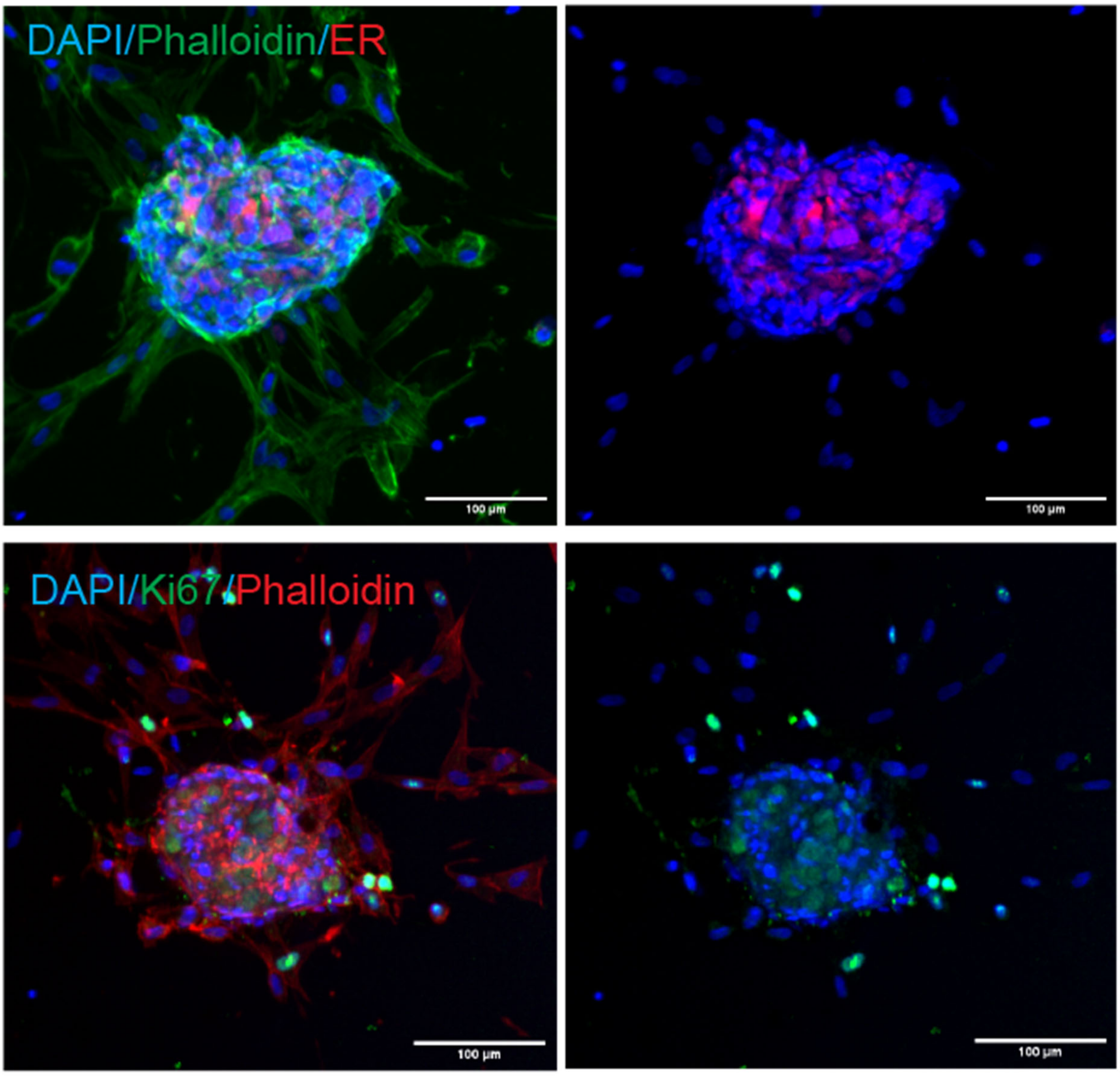

Figure S1. – Evidence of Estrogen Receptor and Proliferative Marker Ki67 Expression in CBCa51 Triple-Positive Breast Cancer Primary Cell Culture.

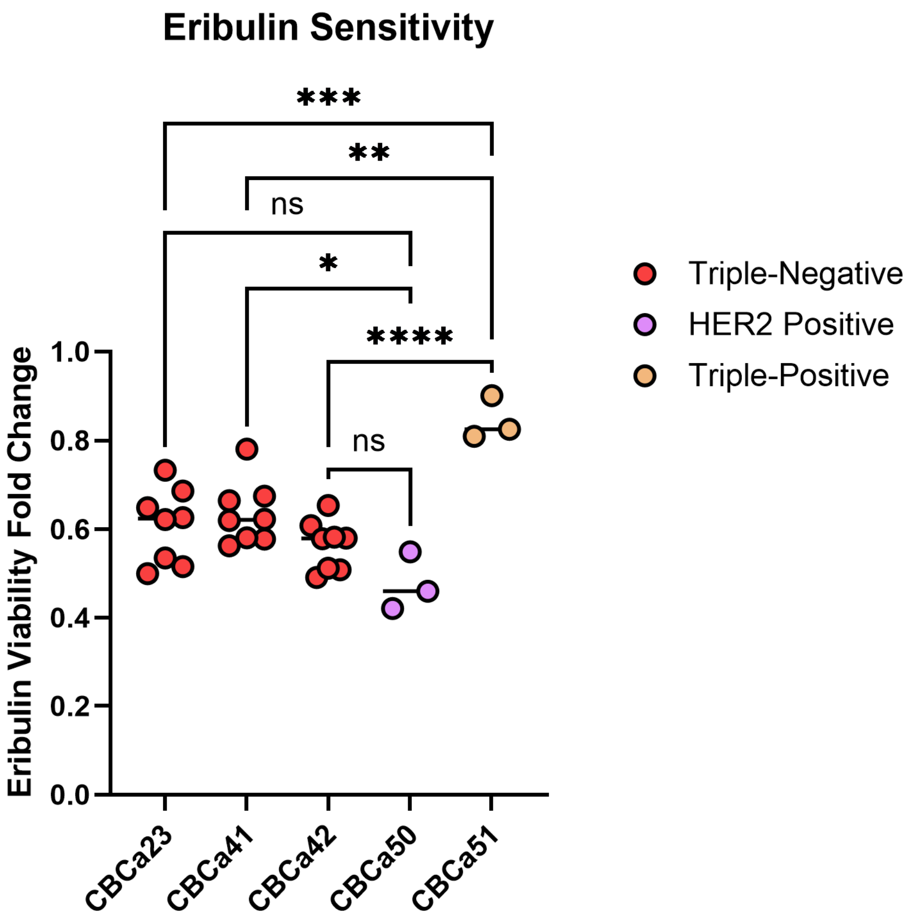

Figure S2. – Statistical Comparison of Primary Patient-Derived Cell Responses to Eribulin Treatment Denoted by Subtype. Fold change of CellTitreGlo-based cell viability is shown following 7 days of 0.5 nM of eribulin exposure. Data is derived Figure 4a to show statistical differences between molecular subtypes regarding eribulin sensitivity. Significance determined by One-Way ANOVA with Dunnett's Multiple Comparison Test ns – not significant, *$p < 0.05$, **$p < 0.01$, ***$p < 0.001$ ****$p < 0.0001$.
